# Vitality surveillance at distance using thin-film tandem-like narrowband near-infrared photodiodes with light-enhanced responsivity

**DOI:** 10.1126/sciadv.adf9861

**Published:** 2023-02-17

**Authors:** Riccardo Ollearo, Xiao Ma, Hylke B. Akkerman, Marco Fattori, Matthew J. Dyson, Albert J. J. M. van Breemen, Stefan C. J. Meskers, Wijnand Dijkstra, René A. J. Janssen, Gerwin H. Gelinck

**Affiliations:** ^1^ Molecular Materials and Nanosystems, Institute for Complex Molecular Systems, Eindhoven University of Technology, P.O. Box 513, 5600 MB Eindhoven, Netherlands.; ^2^ TNO at Holst Centre, High Tech Campus 31, 5656 AE Eindhoven, Netherlands.; ^3^ Integrated Circuits, Departments of Electrical Engineering, Eindhoven University of Technology, P.O. Box 513, 5600 MB Eindhoven, Netherlands.; ^4^ Dutch Institute for Fundamental Energy Research, De Zaale 20, 5612 AJ Eindhoven, Netherlands.

## Abstract

Remote measurement of vital sign parameters like heartbeat and respiration rate represents a compelling challenge in monitoring an individual’s health in a noninvasive way. This could be achieved by large field-of-view, easy-to-integrate unobtrusive sensors, such as large-area thin-film photodiodes. At long distances, however, discriminating weak light signals from background disturbance demands superior near-infrared (NIR) sensitivity and optical noise tolerance. Here, we report an inherently narrowband solution–processed, thin-film photodiode with ultrahigh and controllable NIR responsivity based on a tandem-like perovskite-organic architecture. The device has low dark currents (<10^−6^ mA cm^−2^), linear dynamic range >150 dB, and operational stability over time (>8 hours). With a narrowband quantum efficiency that can exceed 200% at 850 nm and intrinsic filtering of other wavelengths to limit optical noise, the device exhibits higher tolerance to background light than optically filtered silicon-based sensors. We demonstrate its potential in remote monitoring by measuring the heart rate and respiration rate from distances up to 130 cm in reflection.

## Teaser

A detector for invisible light measures a person’s heartbeat and respiration rate contactless beyond one meter distance.

## INTRODUCTION

Unobtrusive, continuous monitoring of a patient’s vitality is important in health diagnostics and would be facilitated by devices that can be used at the point of care. One of the most valuable and widely tracked vital signs is the heart rate, which can be measured, nowadays, noninvasively via electrocardiogram, ballistocardiography (*1*), photoplethysmography (PPG) (*2*), and thoracic motion tracking (*3*). Optical techniques, such as PPG and thoracic motion tracking, rely on the interaction of light with the human body and thus enable a remote, i.e., at distance, and entirely contactless assessment of the cardiorespiratory activity. This ensures more hygiene than on-skin devices and is highly desirable when monitoring newborns and people with skin conditions or when more comfort for the patient is required, such as during sleeping or resting time.

By sensing the volumetric variations of arterial blood in time as light oscillations, i.e., extracting a PPG signal, parameters such as heart rate, heart rate variability, and saturated blood oxygenation can be derived. This is carried out by illuminating subcutaneous tissues with a green, red, or near-infrared (NIR) light source followed by the detection of transmitted or back-reflected light with a photodetector. For remote monitoring, a NIR light source is preferred, as it is invisible and safer to the human eye (*4*), especially for prolonged exposure times of continuous monitoring but requires high NIR sensitivity from the photodetector. With increasing distances, challenges arise: Signals inevitably become weaker and have lower integrity because of optical losses and uncontrolled fluctuations of ambient light, which raises the background noise. Resolving these complications is thereby an essential step toward the next generation of remote sensing devices. Commercial infrared cameras with advanced acquisition algorithms have been successfully used (*5*–*7*), but this approach relies on collecting light onto a small surface using expensive bulky optics that complicates their compactness and unobtrusive integration in everyday objects, such as bed mattresses, desk chairs, and seat cushions. In addition, camera-based remote PPG typically analyzes face images, which may imperil the individual’s privacy (*8*). Here, solution-processed thin-film NIR photodetectors that can be processed cost effectively over large area on flexible, lightweight substrates have a clear advantage.

Solution-processed thin-film photodiodes (PDs) have seen tremendous progress in recent years. Several groups have investigated their use in direct-contact PPG (*9*–*15*). Organic PDs (OPDs) and, more recently, perovskite PDs (PPDs) have dominated the scene of this class of devices, bringing high photogeneration sensitivities, fast response times, and low noise levels (*16*–*20*). In addition, OPDs and PPDs exhibit tunable bandgaps, which can be controlled from the ultraviolet (UV) to the NIR via material composition and leveraged to vary the type (broad- and narrowband) of spectral sensitivity. For instance, NIR-sensitive PDs have been demonstrated by blending narrow-bandgap polymers in bulk heterojunctions (BHJs) (*21*–*23*) or by introducing tin (Sn) in lead halide perovskites (*24*–*27*). PDs with narrow or multiwavelength responsivity have been achieved without the use of optical filters, by using narrowband organic (*28*–*31*) and perovskite absorbers (*32*), by using the antibatic responsivity of thick active layers (*33*–*36*), or by constructing hybrid perovskite/organic hierarchical structures that self-filter parts of the visible spectrum (*37*–*40*).

While these features hold great promise, discriminating weak NIR signals from ambient optical noise, i.e., high signal-to-(optical) noise ratio, is essential for remote monitoring. This implies that high photoresponsivity to vitality-relevant NIR wavelengths should be combined with a spectrally selective narrowband responsivity, thus eliminating a large part of the background light. Currently, however, filterless solution-processed narrowband PDs feature relatively low quantum conversion efficiencies, especially in the NIR region (*33*, *35*, *37*, *38*, *41*–*44*), and, to date, are still outperformed by optically filtered commercial Si. This restricts their use in several applications, including remote sensing.

Here, we report a self-filtering solution-processed PD having a narrowband and enhanced NIR responsivity that can be used to monitor heartbeat and respiration remotely. The PD is based on a perovskite-organic tandem-like architecture. It is obtained by stacking a narrow-bandgap BHJ film (PM6:Y6) directly on a wider-bandgap perovskite [FAMAPbI_3_, with FA (formamidinium) and MA (methylammonium)] semiconductor with an electron-blocking film (poly{9,9-bis[3’-(*N*,*N*-dimethyl)-*N*-ethylammoinium-propyl-2,7-fluorene]-*alt-*2,7-(9,9-dioctylfluorene)}dibromide [PFN-Br]) as interlayer (structures of PM6, Y6, and PFN-Br are shown in fig. S1). Such a tandem-like architecture results in a narrowband spectral response with external quantum efficiency (EQE) peaking to 70% at 850 nm. When, however, exposed to additional green light, EQE values exceeding 200% are achieved. We show that such exceptional and adjustable NIR sensitivity facilitates the measurement of extremely weak light signals, thus enabling low-illumination vitality monitoring for reduced power consumptions. We then experimentally demonstrate remote heart rate and respiration detection from practical distances up to 130 cm. The PD also exhibits a higher filtering capability and, thus, lower susceptibility to optical noise than a broadband Si PD with optical filters. With this approach, a solution-processable device for remote heart rate and respiration monitoring is provided, paving the way to more dedicated and integrated vitality monitoring applications.

## RESULTS

### Tandem-like perovskite-organic PDs

**Fig. 1.**
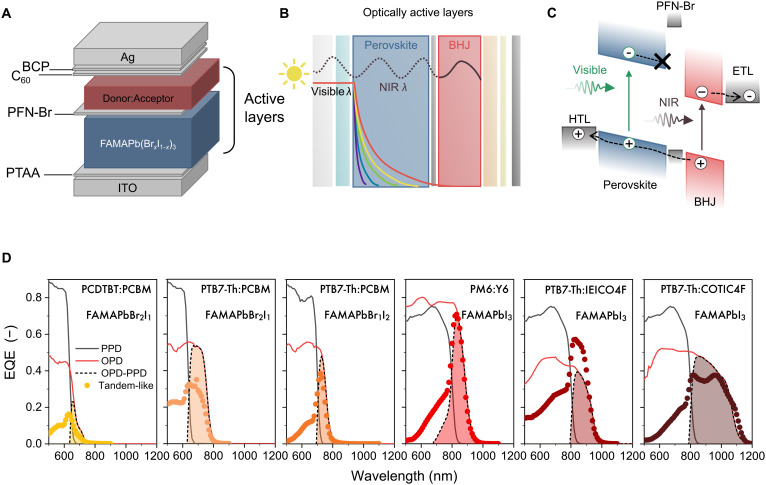
Tandem-like device architecture, working principle, and materials. (**A**) Schematics of PD architecture. (**B**) Illustration of optical field distributions for visible and NIR wavelengths in the two optically active layers based on FAMAPbI_3_ visible absorber and PM6:Y6 NIR absorber. For visible λ up to 650 nm, photons are predominantly absorbed by the perovskite film, while, for NIR λ (850 nm), photon absorption occurs within the organic BHJ layer and is subjected to cavity effects. (**C**) Schematics of the visible wavelengths filtering the tandem-like PD. HTL and ETL stand for hole and electron transport layer, respectively. The collection of photocarriers generated in the perovskite film is blocked by the perovskite/PFN-Br interface. (**D**) EQE as a function of wavelength (colored circle) of tandem-like devices using different perovskite/BHJ combinations, as indicated in the legend. EQE spectrum is compared with that of single PPD (black line) and single OPD (red line) and with the difference of their EQE shapes (dotted black line with colored area).

The architecture and the working principle of the hybrid tandem-like PD are shown in Fig. 1. The device is based on two photoactive layers stacked on top of each other. Between these layers, we purposely deposited an optically inactive thin PFN-Br interlayer, the role of which will be discussed below. The active layers are made of a wider-bandgap perovskite layer combined with a narrow-bandgap organic BHJ consisting of a blend of donor and acceptor organic semiconductors, as schematically shown in Fig. 1A. All active layers are deposited by spin coating and sandwiched between a transparent front indium tin oxide/poly[bis(4-phenyl)(2,4,6-trimethylphenyl)amine] (ITO/PTAA) electrode and a reflective back electrode [C_60_/bathocuproine (BCP)/Ag], resulting in a bottom-illuminated device. The device configuration resembles that of a tandem solar cell, albeit lacking a recombination junction, and will be referred to as tandem-like.

High-energy photons entering the diode via the transparent contact will be almost completely absorbed by the perovskite semiconductor, while low-energy photons are passed to the organic BHJ, where they can be absorbed depending on the BHJ bandgap (see Fig. 1B). By tuning the optical absorption edge of the two individual photoactive layers, it is possible to realize a narrowband NIR spectral response. This requires synergy between the photoactive layers and the PFN-Br interlayer, which plays a critical role in selectively blocking the collection of negatively charged photocarriers generated in the perovskite film. PFN-Br hinders the transport of electrons while facilitating the transport of holes (*45*). As a consequence, positive photocarriers generated in the BHJ layer are effectively collected at the electrodes, producing a photocurrent (Fig. 1C). A similar strategy has been reported before (*39*). The narrowband EQE spectrum is thus the effect of the photocurrent generated in the BHJ because of the longer wavelength absorption of the incident light. In first approximation and assuming unit internal quantum efficiency, the EQE spectrum of the tandem-like device corresponds to the difference between the EQE spectra of the OPD and PPD, i.e., max(EQE_OPD_ − EQE_PPD_, 0).

The location of the narrowband spectral window can be modified by material design. To demonstrate the versatility of this approach, we fabricated tandem-like PDs using mixed-halide Pb-based perovskite films with varied composition in combination with several organic donor-acceptor (D-A) combinations (Fig. 1D). Specifically, FA_0.66_MA_0.34_Pb(Br*_x_*I_1−*x*_)_3_ was used as perovskite, with *x* = 0, 0.33, and 0.66, corresponding to bandgap energies (*E*_g_) of approximately 1.56, 1.80, and 1.95 eV. For the BHJ layer, we used three donor polymers, i.e., poly[*N*-9'-heptadecanyl-2,7-carbazole-alt-5,5-(4',7'-di-2-thienyl-2',1',3'-benzothiadiazole)] (PCDTBT), PTB7-Th, and PM6, blended with four different acceptor materials, i.e., PC_61_BM, Y6, IEICO4F, and COTIC4F, giving five different BHJs with bandgap edges ranging from 700 to 1200 nm. The chemical structures of the organic donor and acceptors are displayed in fig. S1. Single OPDs and PPDs were made for reference. The detailed description of the device fabrication is provided in Materials and Methods.

Figure 1D shows the EQE spectra of the five tandem-like PDs based on different perovskites and D-A BHJs. The measured narrowband EQE spectra of the tandem-like device (colored circles) can be constructed from the EQE spectra of the single-diode devices, i.e., max(EQE_OPD_ − EQE_PPD_, 0), which are plotted for comparison (black, red, and dashed lines). This series shows how both the spectral position and width of the narrowband peak can be easily controlled from the visible to the NIR (1200 nm) and tailored for the application of interest, without any additional optical filter. The low asymmetrical shoulder in the shorter λ region that is visible in some tandem-like devices is due to incomplete light filtering by the perovskite film. The transmitted light is absorbed in the BHJ, causing a small photocurrent at these wavelengths. Further variations may also arise from different light interference in the device (*46*).

Among them, we focus our attention on the FAMAPbI_3_ − PM6:Y6 combination, as it displays a narrow EQE peak [full width at half maximum, <100 nm (*47*)] that is centered on 850 nm, which is a typical NIR wavelength used in PPG measurements. FAMAPbI_3_ has been previously used in extremely sensitive PDs with ultralow dark currents (*24*). The PM6:Y6 blend has demonstrated excellent performance as non-fullerene active material in high-performance organic solar cells (*48*–*50*).

### FAMAPbI_3_ − PM6:Y6 PD characterization

**Fig. 2.**
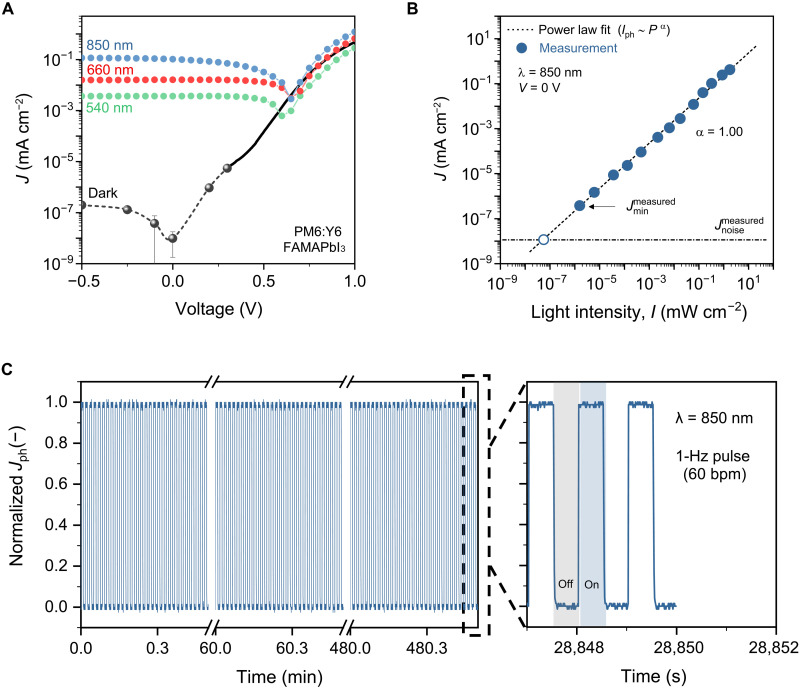
Performance of narrowband PDs based on tandem-like architecture with FAMAPbI_3_ and PM6:Y6 active layers. (**A**) *J*-*V* characteristics in the dark and under low light intensity illumination (0.5 mW cm^−2^) for different wavelengths; in the dark, solid circles are current density values derived from constant voltage measurements over time at discrete biases (fig. S2). (**B**) Linearity plot measured at 0 V showing *J*_ph_ as a function of NIR light intensities (850 nm). (**C**) Continuous tracking over 8 hours of normalized transient photocurrent response of the device upon 850-nm light pulses of 1 Hz, corresponding to 60 bpm, a typical resting heartbeat.

The current density–voltage (*J*-*V*) characteristics recorded in the dark and under illumination for three different wavelengths, i.e., 540, 660, and 850 nm, are shown in Fig. 2A. To exclude capacitive contributions (*24*), we determined the dark current density (*J*_D_) in the range of −0.5 V to +0.3 V by measuring the current over time under constant applied voltages. We consider the latter measurement a more accurate way of determining *J*_D_. The reverse *J*_D_ at *V* = −0.5 V is 2 × 10^−7^ mA cm^−2^, and slowly decreases to 1 × 10^−8^ mA cm^−2^ when approaching *V* = −0.1 V. When illuminated with 0.5 mW cm^−2^ monochromatic 540-nm light, the photocurrent (*J*_ph_) is low at ~4 × 10^−3^ mA cm^−2^, while at 660 and 850 nm (and same light intensity), *J*_ph_ is 1.5 × 10^−2^ and 1 × 10^−1^ mA cm^−2^, respectively, in agreement with the narrowband EQE profile. Notably, *J*_ph_ does not change much whether the device is reverse-biased (−0.5 V) or short-circuited (0 V). Considering the negligible bias dependence of the photocurrent, the device can be efficiently operated at *V* = 0 V.

At the PPG-relevant λ of 850 nm, the tandem-like PD exhibits a close to linear light intensity dependence of *J*_ph_ (at 0 V), with a fitted slope of α = 1.00 (according to *J*_ph_ ~ *I*^α^) and a minimum detected light intensity of 1 nW cm^−2^. This value is close to the minimum light intensity that our equipment can produce repeatedly and consistently at 850 nm. The recorded current noise spectral density (*i*_n_) of the device measured at 0 V is frequency independent (*f* = 1 to 100 Hz) and as low as 9 × 10^−15^ A Hz^−1/2^ (fig. S3). At *V* = −0.5 V, *i*_n_ remains frequency-independent and increases slightly to ∼1 × 10^−14^ A Hz^−1/2^. By considering the measured resulting from noise as the lowest *J*_ph_, the linear dynamic range (LDR), i.e., the span of light intensities within which the device output is linear to the incident light intensity, is >150 dB. At the highest end of light intensity range (1.5 mW cm^−2^), no clear deviation from linearity is observed (Fig. 2B). The excellent linear response over a large NIR light intensity range is advantageous for measuring PPG remotely, as will be shown below.

The stability of the tandem-like PD over time was studied by illuminating the device for >8 hours with light pulses of λ = 850 nm and *f* = 1 Hz to mimic a real-life monitoring of a typical heartbeat at rest, i.e., 60 beats per minute (bpm), and of a duration corresponding to the recommended sleep time for a healthy adult (Fig. 2C) (*51*). No signs of degradation of the photoresponse were observed after the test period, indicating a promising operational stability and reliability to continuous tracking. With 5.6- and 6.05-μs rise and decay times (fig. S4), the device is sufficiently fast for accurately tracking the PPG waveform.

### Enhanced NIR sensitivity by green light illumination

**Fig. 3.**
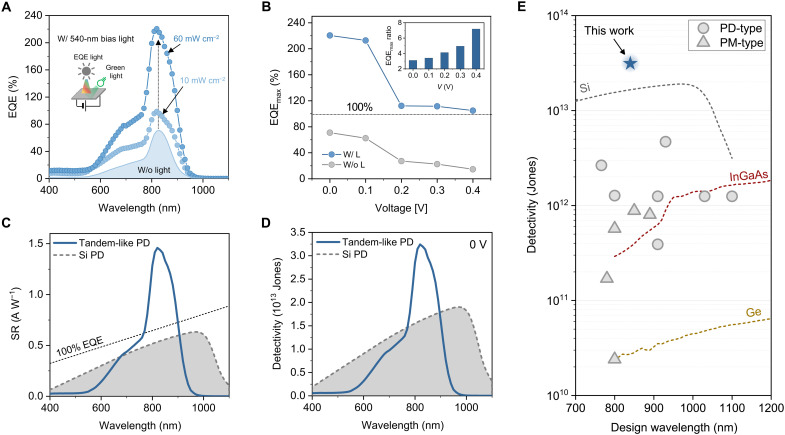
Enhancement of NIR sensitivity by green light illumination. (**A**) EQE as function of wavelength measured with and without additional green (540 nm) light illumination, showing an enhancement in the NIR region. Green light intensities are indicated in the legend. (**B**) Corresponding EQE max (∼830 nm) obtained with and without green illumination plotted as a function of applied bias voltage. For comparison, EQE_max_ under the additional 60 mW cm^−2^ 540-nm light is considered. The inset shows the relative enhancement of EQE due to the additional green light as a function of applied bias expressed as the ratio of EQE_max_ with and without green light. Data are extracted from EQE spectra (fig. S6). (**C**) Spectral responsivity (SR) of tandem-like PD (under 60 mW cm^−2^ 540-nm illumination) compared to that of commercial Si PD. Colored circles indicate the SR at 850 nm for our device, Si and ideal PDs having 100% EQE. (**D**) Detectivity at different wavelengths measured at 0 V for tandem-like PD under additional green light (blue solid line) and for commercial Si reference PD (*i*_n_ = 1.2 × 10^−14^ A Hz^−1/2^) (gray dashed line). Graph in semilogarithmic scale is provided in fig. S10. (**E**) Comparison of noise current–based specific detectivity (D^*^) of our device with state-of-the-art NIR narrowband solution–processed photodetectors (both PD and photomultiplication types, indicated in the legend as PD-type and PM-type, respectively) and with main commercial inorganic PDs, namely, Si (Thorlabs, FDS100-CAL), Ge (Thorlabs, FDG03-CAL), and InGaAs (Thorlabs, FGA21-CAL). The comparison focuses on reported devices with design wavelength lying in the NIR region between 700 and 1200 nm. Further details are provided in fig. S10.

The FAMAPbI_3_ − PM6:Y6 tandem-like PD showed a maximum EQE of 70% at 0 V (Fig 1C). At −1 V, the maximum of the EQE peak (EQE_max_) increases to 80% (fig. S5), confirming the minor reverse bias dependence of the photoresponse. When the tandem-like PD is illuminated with an additional green light source (λ = 540 nm), as schematically represented in Fig. 3A, EQE_max_ increases sharply. For a (green light) intensity of 10 mW cm^−2^, EQE_max_ is almost 100%, while for 60 mW cm^−2^, it reaches 220%. This is exceptional for a PD characterized by low *J*_D_ and noise. Such light effect is also observed under a forward applied bias, where a reduction of the photoresponse, and thus of EQE, is expected (see Fig. 2A). For instance, at *V* = 0.4 V, the lower EQE_max_ of 15% increases to 105%, as shown in Fig. 3B. This corresponds to a lower absolute EQE_max_ value but higher relative enhancement (Fig. 3B, inset). Notably, the narrowband spectral response is retained, as shown in Fig. 3A (fig. S6).

We attribute this strong increase in EQE to above 100% to the transfer and collection of electrons photogenerated by the additional green light in the perovskite film to the BHJ and then to cathode because of a local energy barrier lowering at the PFN-Br interface. The event is triggered by the NIR-generated holes in the BHJ that are approaching the PFN-Br interface from the organic layer side. Therefore, the PFN-Br interlayer acts as an optoelectronic “valve” for electron extraction, and the incident NIR photons can open this valve in a way that resembles what occurs in a photomultiplication (*52*). The extent of this process varies as a function of the intensity of the additional green light, with which the NIR photoresponsivity of the device can therefore be regulated. A schematic illustration of the mechanism with a more detailed description is provided in fig. S7. As a control experiment, we fabricated a FAMAPbI_3_ − PM6:Y6 PD without the PFN-Br interlayer. As shown in fig. S8, such a device shows neither the strong EQE enhancement nor the narrowband EQE profile, confirming the key role of the PFN-Br layer.

At its maximum enhancement, i.e., at *V* = 0 V and under 60 mW cm^−2^, the narrowband spectral responsivity (SR) is ~1.5 A W^−1^ at 850 nm (Fig. 3C). At this wavelength, the tandem-like PD is almost three times more responsive than a commercial Si diode and two times more than an ideal PD with 100% EQE. Notably, such responsivity remains unaltered even 6 months after the fabrication of the device (fig. S9), indicating a long shelf-life. By calculating the specific detectivity from the measured noise current in dark condition using D^*^ = SR (*AB*)^1/2^ *i*_n_^−1^, with *A* = 0.04 cm^2^ and *B* = 1 Hz, we achieved a peak D^*^ of ∼3 × 10^13^ Jones at 850 nm (Fig. 3D), which surpasses that of inorganic Ge, InGaAs, and, notably, Si and is among the highest reported for NIR solution-processed PDs (*53*, *54*). The device noise level under modulated green light remains largely unaltered (fig. S10). Our device excels, in particular, when compared with state-of-the-art narrowband photodetectors (both PD and photomultiplication types) with designed wavelength, i.e., peak of narrowband spectrum, in the NIR (Fig. 3E). Overall, the narrowband ultrahigh NIR sensitivity meets all requirements to ensure a high signal-to-(optical) noise ratio.

### Enhanced NIR sensitivity for detection of extremely weak PPG signals

In a PPG measurement, high incident light intensities are generally preferred because of the severe light attenuation in the fingertip by skin structures (muscles, dermis, bones, tissues, and veins), often resulting in drops >90%, as we show in fig. S11. Considering that only a small portion (∼1%) of the attenuated light contains information related to the cardiac activity, measuring PPG using weak (and low-powered) light sources can be prohibitive and lead to inconclusive readings. Enhancing the NIR sensitivity can help overcome this limitation. To show this, we performed low-illumination PPG measurements corresponding to light intensity variations lower than 1 nW cm^−2^, which is close to the minimum detectable light intensity of the device (Fig. 2B).

**Fig. 4.**
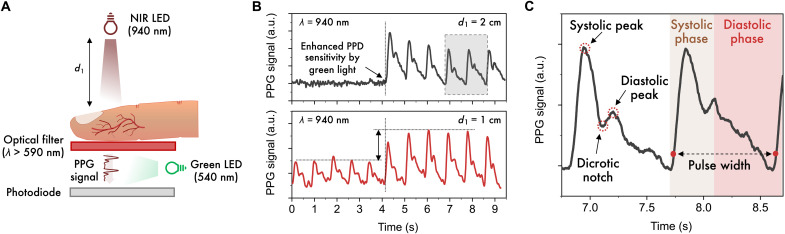
Detecting weak PPG signals using the tandem-like PD with enhanced NIR sensitivity. (**A**) Schematic overview of the experimental conditions under which a PPG signal has been measured by the tandem-like PD with enhanced NIR sensitivity by green light illumination. As NIR light source, we used a LED emitting at 940 nm, i.e., at the edge of device SR, located at 1 or 2 cm above the finger. The PD was place instead ∼1.5 cm below the finger, while the green LED (540 nm) was placed within this gap. In addition, an optical long-pass filter (λ > 590 nm) was placed underneath the finger to prevent unwanted PPG signal generation from the direct interaction of green light with the latter. (**B**) PPG signal measured in transmission from the finger as described in (A) without and with enhanced NIR sensitivity enabled by green light illumination. For the experiment, the green LED was driven at 1.5 V. (**C**) Enlargement of the PPG waveform indicated by the gray outline in (B), which shows the typical PPG waveform features, such as the systolic and diastolic peaks (and phases), dicrotic notch, and pulse width. a.u., arbitrary units.

In our experiment, we set the initial light intensity at 1.3 μW cm^−2^ so that only 0.8 nW cm^−2^ is modulated by heart-pumped blood flow and relevant for PPG. Additional optical losses occurred because of light-emitting diode (LED)–finger and finger-PD distances, shown schematically in Fig. 4A, where more experimental details are also provided. In particular, two measurements were performed, in which the distance between the LED and the finger was varied from 1 to 2 cm. The PPG setup was configured in such a way to intentionally undermine the already weak light before and after the interaction with the fingertip and obtain a weak or null PPG output signal.

Figure 4B shows the resulting PPG signals measured over time with our device at the two LED distances before and after the additional green illumination was turned on. In the absence of additional green light, i.e., in the first 4 s of the recording, the measured PPGs are relatively poor. In case of the larger LED-finger distance, no PPG signal was detectable at all. A similar result was obtained by using a commercial Si diode (fig. S12). When the green light is switched on and, thus, the sensitivity of the tandem-like PD increases, i.e., *t* > 4 s, full PPG waveforms could be recorded. The pulses measured by the tandem-like PD with enhanced NIR sensitivity show the systolic peak, dicrotic notch, and diastolic peak that typically characterize the rising (anacrotic) and descending (catacrotic) phases related to the variation of blood volume by cardiac dilation (Fig. 4C). From the systolic peak-to-peak interval, a heart rate of 67 bpm was extracted. Collectively, combining low-powered NIR and green LEDs resulted in a total power consumption of ~0.2 mW, which is two orders of magnitude lower than the power required by the single NIR LED (~40 mW) to produce a qualitatively similar PPG waveform. This effect is due to the enhanced responsivity under additional green light and the nonlinear power consumption of the NIR LED when increasing light intensity (figs. S11 and S13).

### Remote vital sign measurements with tandem-like PD

**Fig. 5.**
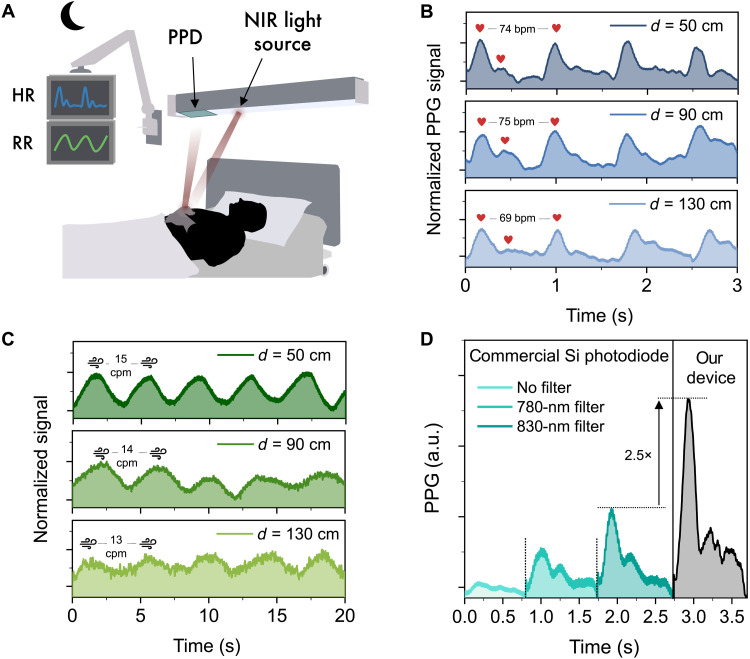
Demonstration of vital sign measurements at distance for constant, wireless monitoring. (**A**) Schematic illustration of the application of the tandem-like PD in a hospital bed for heart and respiration rate monitoring at distance of a patient during sleep/resting time. The optical noninvasive wireless setup that can be integrated in the hospital’s equipment and maximize the comfort of the patient. (**B**) Normalized PPG signal measured at different PD-finger distances using NIR light (850 nm). (**C**) Normalized respiration rate signal measured through clothing at different PD-chest distances using NIR light (850 nm). (**D**) Comparison of PPG signals measured at distance (*d* = 50 cm) under the same experimental conditions using our tandem-like device and a broadband commercial Si PD without and with 780- and 830-nm optical filters to suppress ambient optical noise.

Next, we used the tandem-like PD to measure both the heartbeat and respiration rate remotely. This would, for example, enable unobtrusive vital sign monitoring of a patient in a hospital bed during sleep with more comfort, as schematically proposed in Fig. 5A. To provide a proof of concept, we tracked both heart and respiration rates of a 29-year-old volunteer from three different distances by measuring the PPG signal (for heart rate) and the thoracic motion (for respiration rate) from the individual’s hand and chest, respectively. Both the device and the NIR light source were located at 50, 90, and 130 cm from the body locations, as schematically shown in fig. S14A. A photograph of the experimental setup for heartbeat monitoring is shown in fig. S14B. As NIR light source, we used a LED emitting at 850 nm that matches the responsivity peak of our device. The lower risk and disturbance to the human eye of NIR radiation then allowed us to drive the LED at higher voltage (8 V) for a brighter light intensity to compensate for the longer distances. The precise intensity value interacting with the body parts was, however, hard to estimate because of the wide diffusion angle (35°) of the light beam of the LED and the undefined illuminated area of the body. The measurements were performed indoor during a sunny day with the curtains partially closed to replicate the ambient illumination of a room during a typical daytime sleeping (fig. S14B).

Figure 5B shows the normalized PPG signals measured by the tandem-like PD at the three distances mentioned above, i.e., 50, 90, and 130 cm. At each distance, the systolic and diastolic phases are observed, from which heart rates of 74, 75, and 69 bpm were extracted, respectively. In Fig. 5C, the respiration rates measured at the same distances are shown, resulting in 15, 14, and 13 cpm (cycles per minute), which well lie in the respiratory rate range of a healthy adult at rest (*55*). The variation of reflected light intensity due to respiratory thoracic motion was estimated through clothing. Both measured signals by the device were amplified by a preamplifier and normalized for comparison. More details on the acquisition technique are provided in Materials and Methods.

Notably, no accurate alignment between the LED and the PD was needed to detect the PPG signal at each distance. A rough directing of the LED light and the PD toward the interested acquisition area of the body was sufficient to read both heart and respiration rates. This would impose fewer constraints to future device integration into the individual’s surroundings, enabling multipoints monitoring and better coverage. In addition, several PDs could be fabricated in parallel so that the failure of a single PD would not lead to a failing PPG signal. For completeness, heart rate was also measured in transmissive mode, with the LED located in proximity of the fingertip and in opposite position compared to the PD. In this case, PPG signals were acquired at longer PD-hand distances of 100, 150, and 210 cm (fig. S15).

Last, we compared the optical noise tolerance during remote PPG measurements with that of a broadband commercial Si PD (Thorlabs, FDS100-CAL) equipped with optical filters. Measurements were performed under the same conditions for each device (*d* = 50 cm, reflective mode, same applied voltage to the LED as above). When no optical filter is used, the broadband Si diode hardly discriminates the PPG waveform. Stronger PPG signals are discerned when optical cutoff filters are used, as shown in Fig. 5D. The largest PPG signal (obtained with 830-nm filter) is, however, 2.5× lower than that recorded with our device, proving the higher filtering efficacy of our narrowband device. As a result, PPG measurement at longer distances has not been possible with an optically filtered Si diode.

## DISCUSSION

We have developed a self-filtering solution-processed, thin-film PD with enhanced NIR responsivity for heartbeat and respiration monitoring at distance using a tandem-like structure made of perovskite-organic BHJ active layers and a PFN-Br interlayer. Within this architecture, the stacking of the two active layers simplifies the device integration and enables a filterless narrowband spectral response, which can be tuned from visible to NIR by selecting the appropriate combination of perovskite and organic semiconductor materials. By integrating the composition of interest (FAMAPbI_3_ − PM6:Y6) into a PD, we achieved low dark and noise current, a wide LDR > 150 dB under NIR light (850 nm), and a stable device response over time (>8 hours) to pulsed light. We have demonstrated an enhancement of the device sensitivity to NIR (850 nm) upon additional green light illumination, expressed in terms of EQE values exceeding 200%, SR of 1.5 A W^−1^, and D^*^ of 3 × 10^13^ Jones. This exceptional performance allowed us to detect extremely weak PPG signals resulting from low illumination source. We have also demonstrated remote heart rate and respiration rate monitoring from beyond 130 cm, which shapes a noninvasive, more comfortable monitoring scenario. In addition, in comparison with an optically filtered broadband Si PD, our device exhibits a higher filtering capability and, thus, lower susceptibility to optical ambient noise. These features, coupled with a cost-effective large-area processing, make this tandem-like PD promising for the next generation of contactless vital sign monitoring devices.

## MATERIALS AND METHODS

### Materials

All materials were purchased from commercial sources and used without further purification unless otherwise mentioned. PbI_2_ and PbBr_2_ (99.99%) were purchased from TCI Chemicals. All the organic salts for perovskites were purchased from GreatCell Solar. All the solvents were bought from Sigma-Aldrich. PCDTBT, PTB7-Th, IEICO-4F, COTIC-4F, and PC_61_BM were purchased from 1-Material Inc., while PM6, Y6, and PFN-Br were purchased from Solarmer Materials Inc.

### Active layer deposition

Solution preparation and film deposition were performed in an N_2_-filled glove box. FA_0.66_MA_0.34_Pb(Br*_x_*I_1−*x*_)_3_ was prepared via two-step spin coating according to a previous publication from our group (*56*). For the molar fraction (*x*) of 0 and 0.33, 553.2 mg of PbI_2_ was dissolved in *N*,*N*´-dimethylformamide (DMF; 0.876 ml) and dimethyl sulfoxide (DMSO; 0.0864 ml), while for *x* = 0.66, 276.6 mg of PbI_2_ and 220.2 mg of PbBr_2_ were dissolved in the same amount of DMF and DMSO. The solutions were spin-coated at 3000 rpm for 30 s. Then, the organic solutions made of 53.48 mg of FAI and 25.6 mg of MAI (for *x* = 0), 39.23 mg of FABr, and 17.57 mg of MABr (for *x* = 0.33 and 0.66) in isopropanol (1 ml) were spin-coated at 3000 rpm for 60 s. The perovskite films were thermally annealed at 100°C for 30 min. The PFN-Br interlayer was prepared by dissolving PFN-Br in methanol (0.5 mg ml^−1^) and by spin coating the solution at 3000 rpm for 30 s, followed by a thermal anneal at 90°C for 5 min. PCDTBT:PC_61_BM film was prepared by blending PCDTBT with PC_61_BM (1:4 w/w) in chlorobenzene (CB) (35 mg ml^−1^) and by spin coating the solution at 3000 rpm for 30 s, followed by a thermal anneal at 60°C for 15 min. PTB7-Th:PC_61_BM film was prepared by blending PTB7-Th with PC_61_BM (1:1.5 w/w) in CB (20 mg ml^−1^) and by spin coating the solution at 3000 rpm for 30 s, followed by a thermal anneal at 90°C for 15 min. PTB7-Th:IEICO-4F and PTB7-Th:COTIC-4F films were prepared by blending PTB7-Th with IEICO-4F (1:1.5 w/w) and COTIC-4F (1:1.5 w/w), respectively, in CB (20 mg ml^−1^) and by spin coating the solutions at 3000 rpm for 30 s, followed by a thermal anneal at 90°C for 15 min. PM6:Y6 film was prepared by blending PM6 with Y6 (1:1.2 w/w) in chloroform (16 mg ml^−1^) and by spin coating the solution at 3000 rpm for 30 s, followed by a thermal anneal at 110°C for 10 min.

### PD fabrication

All tandem-like PDs and single PPDs share the same device stack, which was fabricated as follows. Transparent ITO (135 nm) was deposited by sputtering on glass and patterned via photolithography to shape the bottom electrode. On top, a thin SiN (50 nm) layer was deposited and structured via dry etch to define the active area of the ITO electrodes and to prevent leakage currents (*57*, *58*). Next, a 30-min UV-ozone treatment was performed on the substrates, followed by PTAA (3 mg ml^−1^ in toluene) deposition via spin coating at 5700 rpm for 30 s. The layer was then thermally annealed at 100°C for 10 min. C_60_ (20 nm; SES Research), BCP (8 nm; Lumtec), and the Ag top electrode (100 nm) were thermally evaporated under high vacuum (≈10^−7^ mbar).

Single OPDs were fabricated using a different stack. An ITO film (100 nm) was sputtered on glass first, followed by an amorphous indium gallium zinc oxide (IGZO) film (16 nm) deposited via sputtering at room temperature with a metal ratio of 1:1:1. Both the ITO and IGZO were then structured by photolithography. On top, an SU-8 layer was deposited and patterned to cover the perimeter of the electrodes. After the deposition of the active films, 15 nm of MoO_3_ and 100 nm of Ag were thermally evaporated as top contact under high vacuum (≈10^−7^ mbar).

### Tandem-like PD characterization

Dark current density was measured in an N_2_-filled glove box through manual probes connected to an Agilent 4155C semiconductor parameter analyzer. For an accurate determination of *J*_D_, a constant reverse voltage bias was applied over time to eliminate unwanted charging effects. The stability of the device to light pulses over time was measured using a digital oscilloscope (Tektronix, TDS3032B) and NIR light pulses (850 nm) generated by a LED driven by a wave function generator (PeakTech, 4040). Within the same setup, the photocurrent response of the PPD to different light intensity was measured using the same NIR LED, which was calibrated with a reference silicon PD (Thorlabs, FDS100-CAL). The EQE custom-made setup consisted of a tungsten-halogen lamp, a monochromator (Oriel, Cornerstone 130), a chopper, a preamplifier (Stanford Research Systems, SR570), and a lock-in amplifier (Stanford Research Systems, SR830 DSP). All EQE measurements were performed using a lock-in detection and modulated probe light to extract the photocurrent at the modulation frequency and reject all other frequency and continuous components. The devices were transferred in the setup through a N_2_-filled box equipped with a quartz window, on which a circular aperture (diameter, 1 mm) was applied. The additional green light illumination was provided by shining a 530-nm LED toward the device following the same optical path of the EQE light. EQE signal was calibrated with a reference silicon solar cell. The SD of this setup is less than 0.005 electron per photon (in the range 350 to 1050 nm of wavelengths). Noise measurements were performed in a battery-powered current-to-voltage conversion readout circuit developed with off-the-shelf components. The setup is arranged in a metal enclosure to shield the device from electromagnetic interference and keep it in dark conditions. The PD (active area of 4 mm^2^) was connected by means of two probes and triaxial cables to a trans-impedance amplifier (TIA) implemented with the operation amplifier Analog Devices (ADA4530). The device was biased by applying an adjustable DC voltage source to the noninverting terminal of the TIA. The output of the TIA is fed to an active band-pass amplifier (built using Analog Devices AD8065 operational amplifier) and lastly read out by a dynamic signal analyzer (HP35670A).

### Heartbeat and respiration rate measurement setup

Measurements were performed by connecting, in sequence, the PD by means of small probes (integrated in a custom-made three-dimensional printed holder) to a signal amplifier (Stanford Research Systems, SR570), a lock-in amplifier (Stanford Research Systems, SR830 DSP), and an oscilloscope (Tektronix, TDS3032B). Measurements were carried out in ambient conditions, for which a protective barrier was laminated onto the PD. As light sources, two NIR LEDs (940 and 850 nm) and a green LED (540 nm) were powered by a wave function generator (PeakTech, 4040). During the acquisition, raw signals have been electronically filtered using a band-pass digital filter from 0.5 to 10 Hz to remove the out-of-band and non-pulsatile (DC) components. In case of respiration rate monitoring, the signal amplifier (Stanford Research Systems, SR570) was directly connected to the oscilloscope (Tektronix, TDS3032B), without using the lock-in amplifier. In addition, a band-pass digital filter from 0.1 to 10 Hz was used.

## Supplementary Materials

### This PDF file includes:

Figs. S1 to S15 Table S1 Legend for data S1 References
