# Supplementary material for: Vitality surveillance at distance using thin-film tandem-like narrowband near-infrared photodiodes with light-enhanced responsivity
Source: Sci Adv. 2023 Feb 17;9(7):eadf9861. doi: 10.1126/sciadv.adf9861 (PMC9937568; doi:10.1126/sciadv.adf9861)
Supplement: Supplementary file 1 — Figs. S1 to S15 Table S1 Legend for data S1 References [file sciadv.adf9861_sm.pdf]

Science Advances
AAAS

Supplementary Materials for

Vitality surveillance at distance using thin-film tandem-like narrowband near-infrared photodiodes with light-enhanced responsivity.

Riccardo Ollearo *et al.*

Corresponding author: René A. J. Janssen, r.a.j.janssen@tue.nl

Sci. Adv. **9**, eadf9861 (2023)
DOI: 10.1126/sciadv.adf9861

The PDF file includes:

Figs. S1 to S15
Table S1
Legend for data S1
References

Other Supplementary Material for this manuscript includes the following:

Data S1

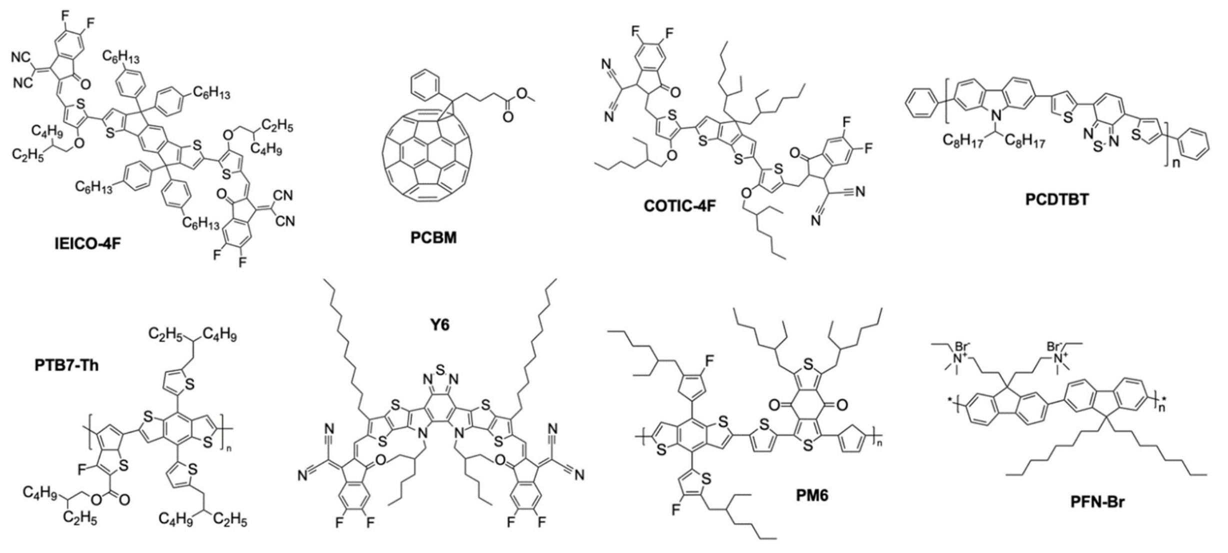

Fig. S1. Chemical structures of the organic donor and acceptor semiconductor materials used in this work. The chemical structure of PFN-Br interlayer is also included.

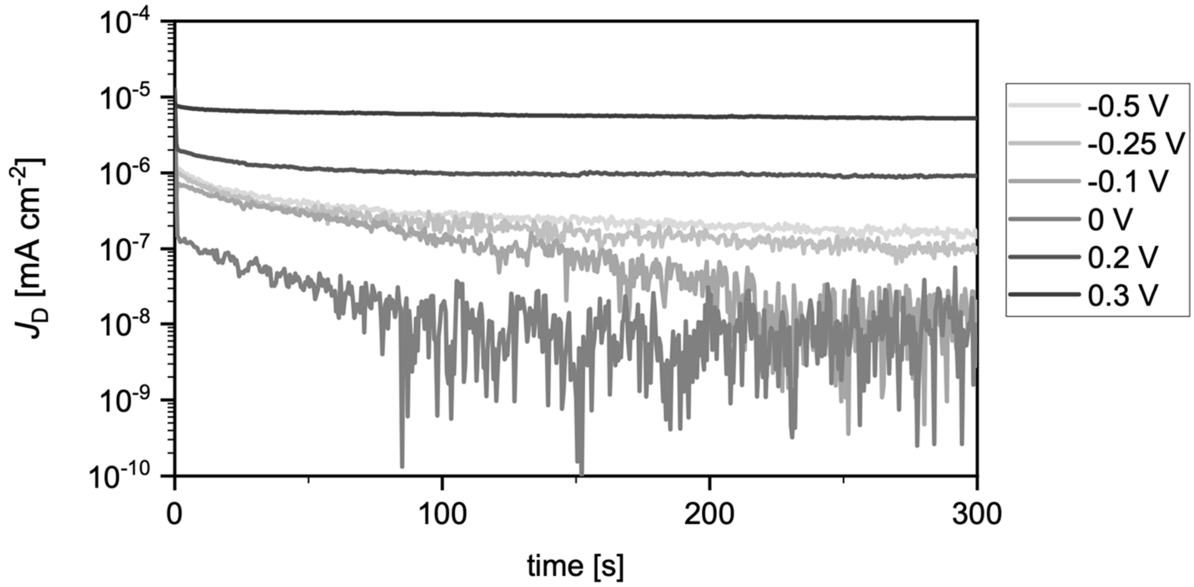

Fig. S2. Time dependence of dark current density. J_D recorded over time by applying a constant bias voltage, as indicated in the legend, for the tandem-like photodiode with $FAMAPbI_3$ and PM6:Y6 active layers.

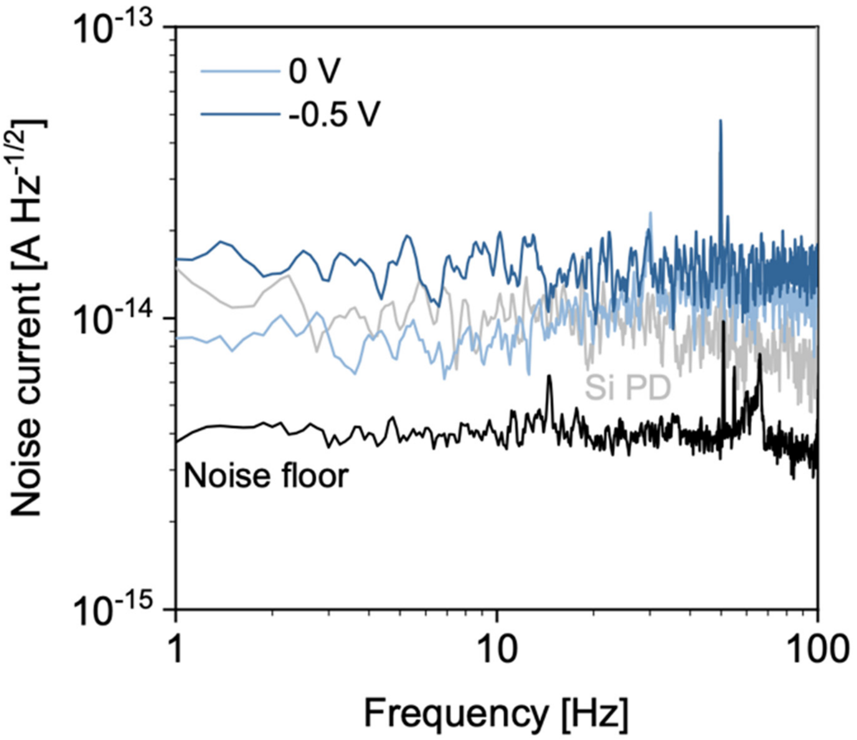

Fig. S3. Noise current spectral density as a function of frequency. Noise current spectral density measured at −0.5 and 0 V for the tandem-like photodiode with $FAMAPbI_3$ and PM6:Y6 active layers. For comparison, the noise of a commercial Si photodiode at −0.5 V is also shown. Each spectrum is obtained with an average of 15 measurements. Harmonics with large amplitude in the spectrum are due to powerline interference. Black line represents the noise floor of the setup (~ 4×10^{-15} A Hz$^{-1/2}$).

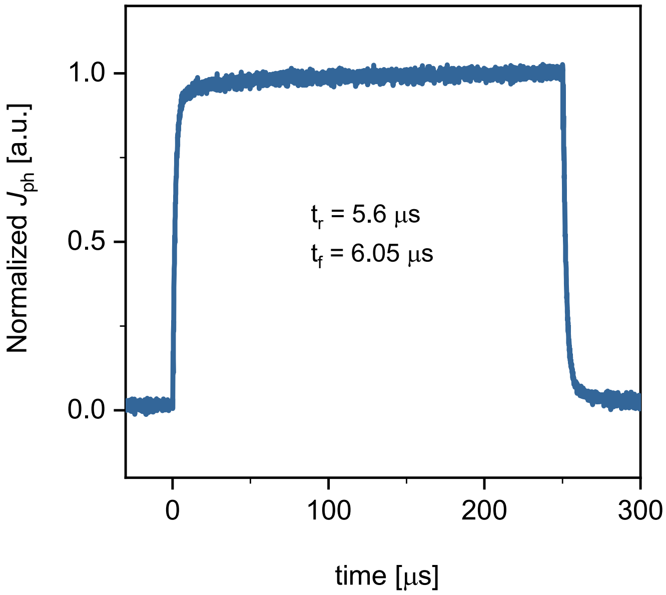

Fig. S4. Normalized transient photocurrent response. Normalized transient photocurrent response upon square light pulses of NIR light (850 nm) of 250 μs duration.

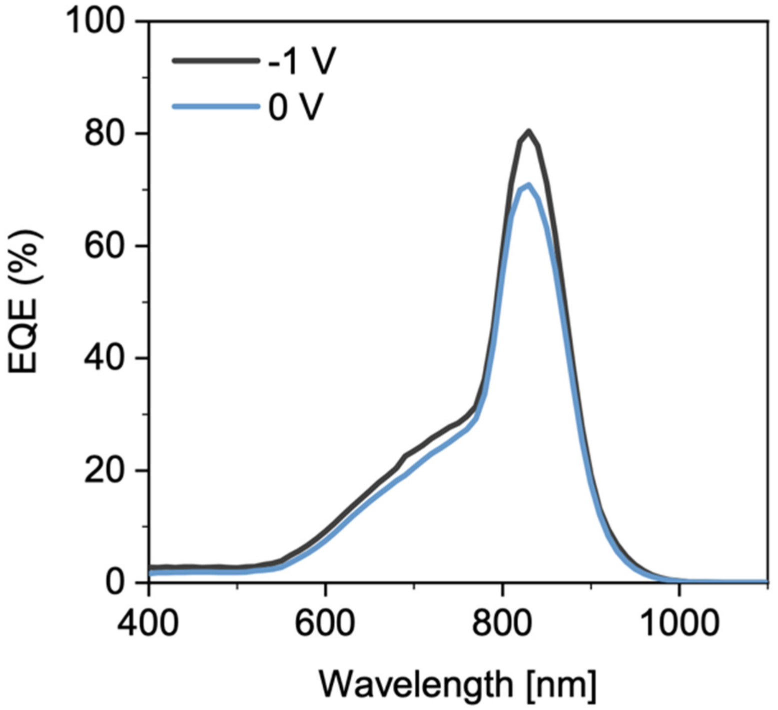

Fig. S5. Voltage dependence of EQE. EQE as function of wavelength measured at 0 and −1 V.

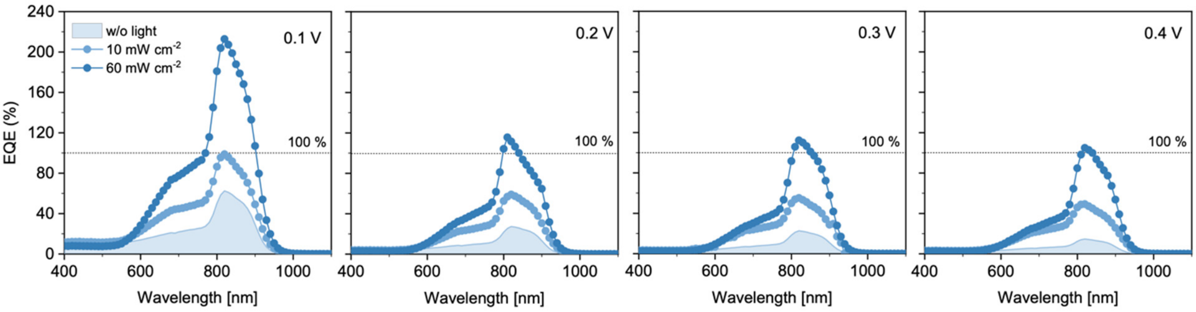

Fig. S6. EQE enhancement as function of applied voltage. EQE as function of wavelength measured with and without additional green (540 nm) light illumination for different applied voltages. Green light intensities are indicated in the legend.

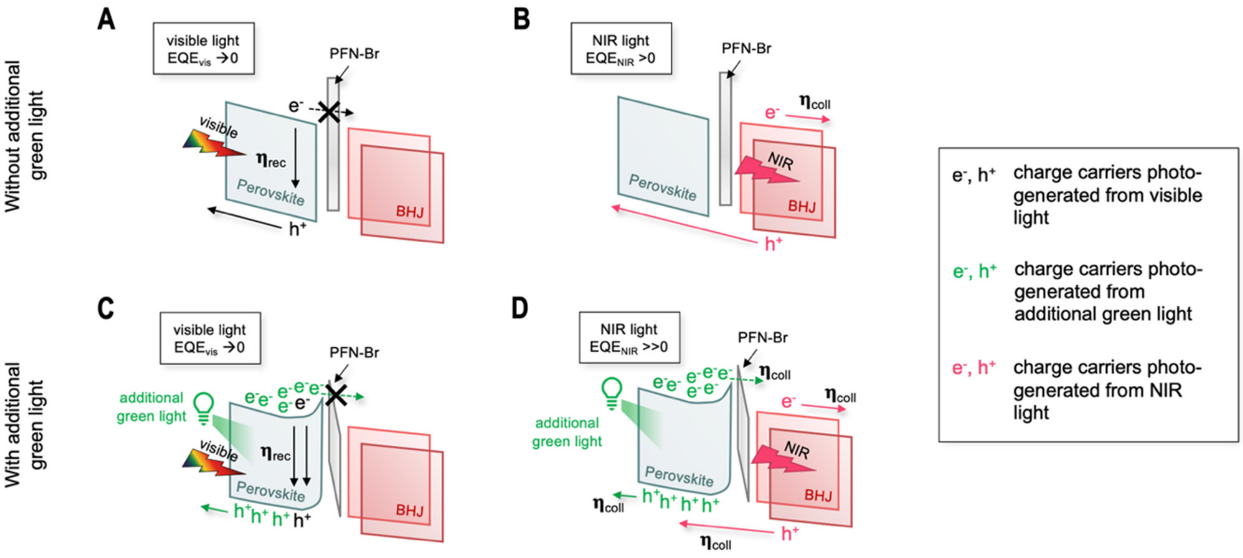

Fig. S7. Schematic illustration of the photophysical mechanisms occurring in the device upon different illumination conditions. (A) Visible light: photogenerated electrons in the perovskite upon absorption tend to accumulate at PFN-Br interface, thus screening the internal electric field that promotes carriers extraction, facilitating instead their recombination. As result, charge collection is largely suppressed and EQE is extremely low, approaching zero. (**B**) NIR light: photogenerated carriers in the BHJ are successfully transported and collected at the electrodes. (**C**) Visible light (with additional green light): the additional green light is entirely absorbed by the perovskite film, where a large population of photo-generated electrons cannot be extracted and piles up at the interface with PFN-Br. This shifts the conduction band of perovskite upward and closer to the LUMO level of PFN-Br, thus redistributing the internal electric field of the latter and lowering the electron-blocking energy barrier. Similar as for (**A**), at the generation of electron and hole pair in the perovskite upon absorption of visible light, charge extraction is hindered. Given the population of photogenerated electrons and holes by additional green light, (visible) electron and holes will more likely recombine, resulting in a very low, almost negligible EQE. (**D**) NIR light (with additional green light): in this situation, the background population of excited electrons and holes leads to EQE to NIR photons exceeding 100%. The additional green light is entirely absorbed by the perovskite film, forming a large population of excited electrons that cannot be extracted and piles up at the interface with PFN-Br. Such accumulated charges shift the conduction band of perovskite upward and closer to the LUMO level of PFN-Br, thus redistributing the internal electric field of the latter and lowering the electron-blocking energy barrier. The extent of this process is regulated by the intensity of additional green light. The incoming NIR light is here absorbed in the BHJ. As described for (**B**), both (NIR) photogenerated electrons and holes can be extracted. However, when the (NIR-) photogenerated holes approach the PFN-Br from the BHJ, the electron-blocking energy barrier further reduces, thus enabling the transfer of a portion of electrons from the perovskite towards the cathode. As a result, from a single NIR photon absorbed by the tandem-like device, more than one carrier is extracted as photocurrent causing an EQE > 100%. The mechanism recalls that of a photomultiplication, but with a lower gain, occurring locally at the junction between the perovskite and the BHJ, due to the PFN-Br interlayer, and is light-activated. Due the complexity

of the system and relative uncertainty of the energetics, the precise mechanism through which electrons passes the barrier, e.g., jumping over or tunnelling through, is not fully understood at this point.

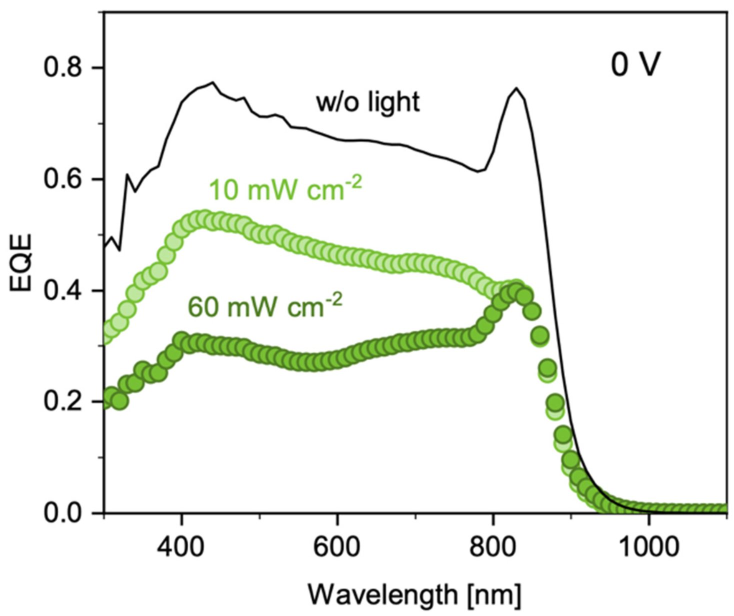

Fig. S8. EQE of the device without PFN-Br interlayer. EQE as function of wavelength measured with and without additional green (540 nm) light illumination for at 0 V for the device without PFN-Br interlayer, showing a broadband spectrum and no enhancement of EQE. Green light intensities are indicated in the legend. Without PFN-Br, all charges generated by additional green light within the perovskite layer can freely move to the BHJ (with the lower bandgap), resulting in a larger population of excited photocarriers in the BHJ. Similar as in the perovskite, the strongly enhanced carrier density in the BHJ, then, results in more electron-hole recombination which reduces the collection efficiency and EQE. This occurs irrespective of where the electron-hole pair is generated, either in the perovskite (from visible photons) or in the BHJ (from NIR photons). Hence, the EQE is reduced over the entire spectrum.

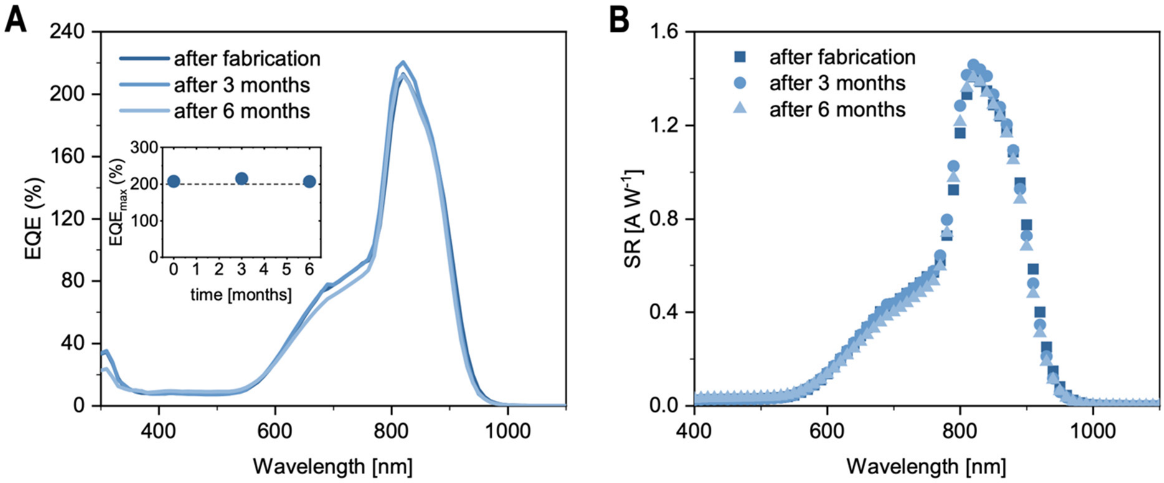

Fig. S9. Device stability. (**A**) Stability of enhanced EQE as function of wavelength measured under additional green light illumination (540 nm, 60 mW cm^{-2}) after fabrication, 3 and 6 months, as indicated in the legend. Inset shows the variation of EQE$_{max}$. (**B**) Stability of the corresponding spectral responsivity.

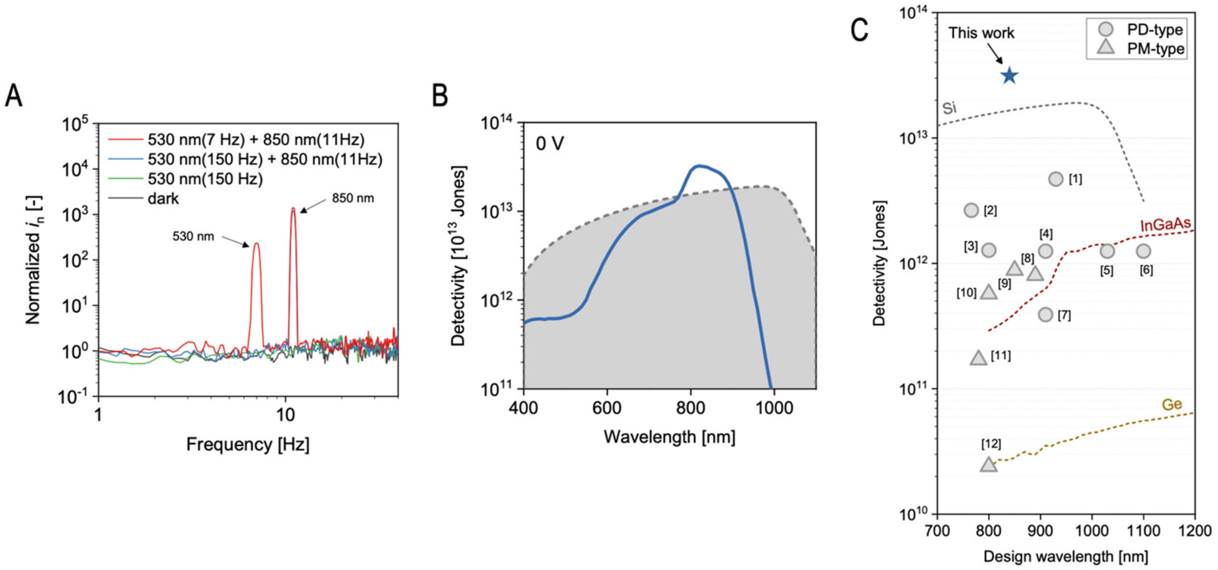

Fig. S10. Noise current and specific detectivity. (**A**) Normalized current noise spectral density (i_n) as function of frequency measured in dark and under 530nm and/or 850 nm light illumination modulated at different frequencies, as described in the legend. Each spectrum has been normalized with respect to that measured in dark. (**B**) Detectivity at different wavelengths as in Fig. 3D in semilogarithmic scale. (**C**) Extended plot of comparison of noise-current based specific detectivity (D^*) of our device with state-of-the-art NIR narrowband solution-processed photodetectors (both photodiode and photomultiplication types, indicated in the legend as PD-type and PM-type respectively). The code number associated to each data point is referenced in Table S1.

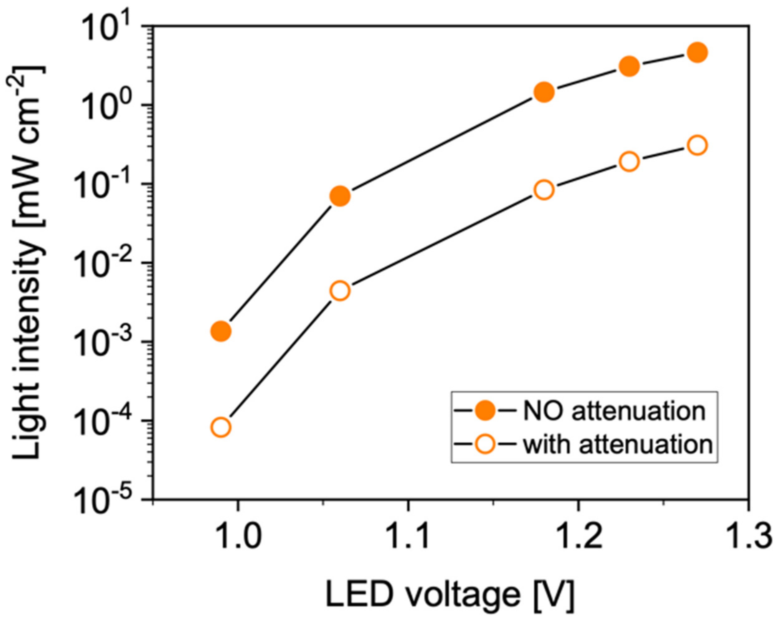

Fig. S11. Effect of LED driving voltage on emitted light intensity. Variation of light intensity as function of LED (940 nm) voltage without and with the attenuation caused by the finger between the LED and the photodiode . For calibration, a commercial Si diode (Thorlabs FDS100-CAL) was used. The measurement has been performed in transmission and by placing the LED at 2 cm from the diode. The attenuated intensity was measured by inserting the finger between the light source and the photodiode.

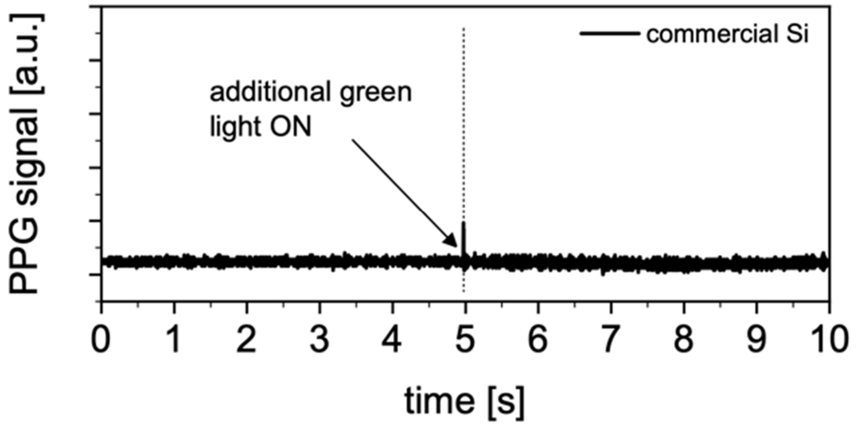

Fig. S12. PPG signal measured with commercial Si photodiode. (Null) PPG signal measured in transmission under the same measurement conditions as described in Fig.4a using a commercial Si photodiode (Thorlabs FDS100-CAL).

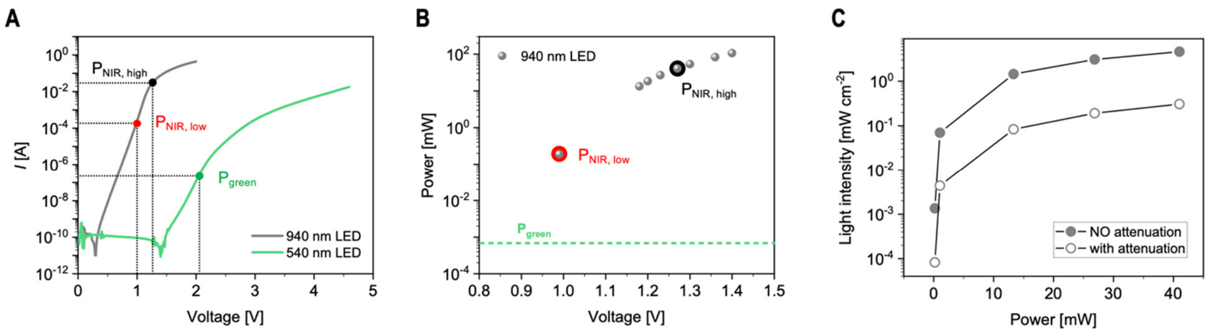

Fig. S13. 940 and 540 nm LEDs characteristics. (**A**) *I-V* characteristics of the green and NIR LEDs. (**B**) Power consumption as function of applied voltage for the 940 nm (NIR) LED, calculated as P = VI. Dashed line shows the value of power used for the additional green LED, while red and black circles indicate the low-power and high-power conditions, respectively, considered in our analysis for the NIR LED. (**C**) Light intensity of the NIR LED as function of power consumption.

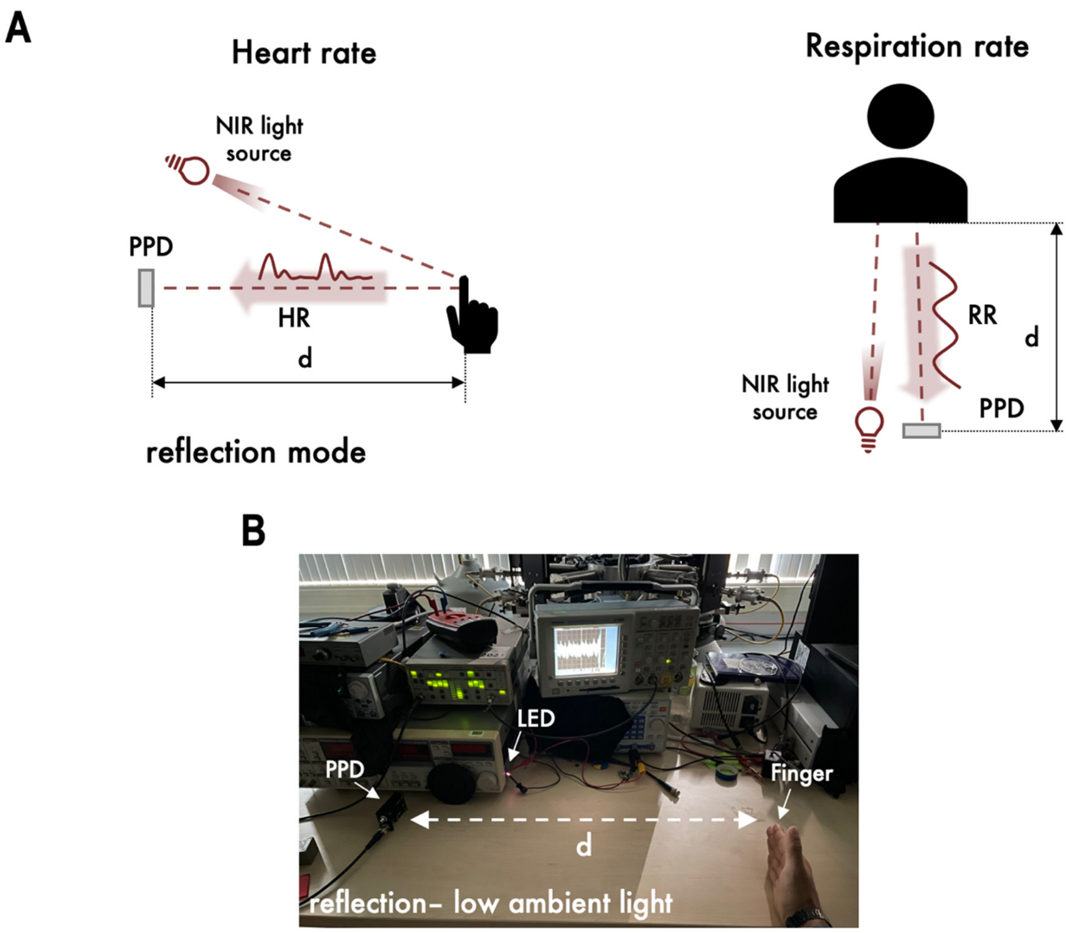

Fig. S14. Experimental setting for heart and respiration rates measurements in reflection. (**A**) Schematic illustration of the experimental set-up used to measure heart rate (left) and respiration rate (right) at distance in reflection. (**B**) Photograph of the experimental setting for remote heart rate monitoring.

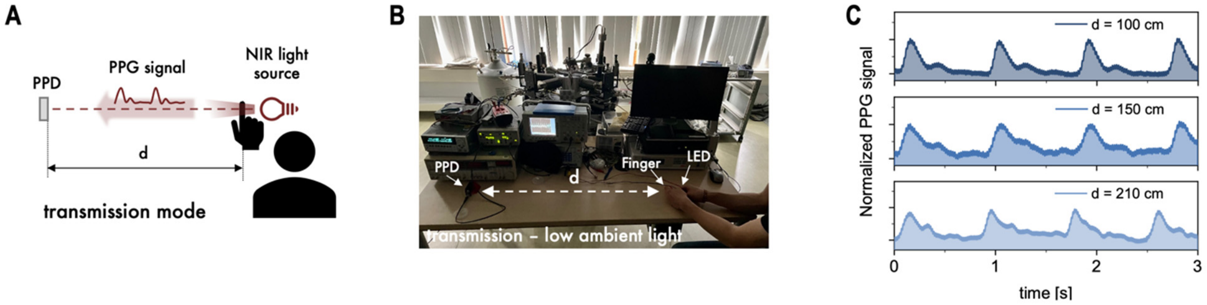

Fig. S15. Experimental setting for heart rate measurement in transmission. (**A**) Schematic illustration and (**B**) photograph of the experimental set-up used to measure the PPG signal at distance in transmission. (**C**) Normalized PPG signal measured at different PD-finger distances using near-IR light (850 nm) in transmission.

Table S1. Comparison of noise-current based specific detectivity (D^*) of state-of-the-art NIR narrowband solution-processed photodetectors as shown in Fig. 3E and Fig. S10.

Code no.	Active material	λ_{design} [nm]	D^* [Jones]	Ref.
1	DPP-DTT:$PC_{71}BM$	930	4.2×10^{12}	(*33*)
2	$MAPbI_3$	766	2.65×10^{12}	(*38*)
3	$MAPbI_3$	800	1.27×10^{12}	(*35*)
4	PTB7-Th:FOIC	910	1.25×10^{12}	(*59*)
5	PTB7-Th:SiOTIC-4F	1030	1.25×10^{12}	(*59*)
6	PTB7-Th:COTIC-4F	1100	1.25×10^{12}	(*59*)
7	D8:C60	910	3.9×10^{11}	(*60*)
8	PDTP-DFBT:$PC_{70}BM$:PbS-QDs	890	8×10^{11}	(*61*)
9	P3HT:PTB7-Th:BEH	850	8.8×10^{11}	(*62*)
10	P3HT:PTB7-Th:$PC_{70}BM$	800	5.7×10^{11}	(*63*)
11	$MAPbI_3$	780	1.7×10^{11}	(*64*)
12	P3HT:PTB7-Th:$PC_{60}BM$	800	2.4×10^{10}	(*65*)

Data S1.
All data shown in the graphs in the manuscript and in the supplementary figures.

REFERENCES AND NOTES

1. O. T. Inan, P.-F. Migeotte, K.-S. Park, M. Etemadi, K. Tavakolian, R. Casanella, J. Zanetti, J. Tank, I. Funtova, G. K. Prisk, M. Di Rienzo, Ballistocardiography and seismocardiography: A review of recent advances. *IEEE J. Biomed. Health Inform.* **19**, 1414–1427 (2015).

2. J. Park, H. S. Seok, S.-S. Kim, H. Shin, Photoplethysmogram analysis and applications: An integrative review. *Front. Physiol.* **12**, 808451 (2022).

3. T. Allsop, G. Lloyd, R. S. Bhamber, L. Hadzievski, M. Halliday, D. J. Webb, I. Bennion, Cardiac-induced localized thoracic motion detected by a fiber optic sensing scheme. *J. Biomed. Opt.* **19**, 117006 (2014).

4. F. C. Delori, R. H. Webb, D. H. Sliney; American National Standards Institute, Maximum permissible exposures for ocular safety (ANSI 2000), with emphasis on ophthalmic devices. *J. Opt. Soc. Am. A. Opt. Image Sci. Vis.* **24**, 1250–1265 (2007).

5. Q. Zhang, Y. Zhou, S. Song, G. Liang, H. Ni, Heart rate extraction based on near-infrared camera: Towards driver state monitoring. *IEEE Access* **6**, 33076–33087 (2018).

6. E. M. Nowara, T. K. Marks, H. Mansour, A. Veeraraghavan, Near-infrared imaging photoplethysmography during driving. *IEEE Trans. Intell. Transp. Syst.* **23**, 3589–3600 (2022).

7. N. Martinez, M. Bertran, G. Sapiro, H. Wu, Non-contact photoplethysmogram and instantaneous heart rate estimation from infrared face video, in *2019 IEEE International Conference on Image Processing* (IEEE, 2020).

8. W. Wang, A. C. Den Brinker, G. De Haan, Single-element remote-PPG. *I.E.E.E. Trans. Biomed. Eng.* **66**, 2032–2043 (2019).

9. N. Cui, Y. Song, C. H. Tan, K. Zhang, X. Yang, S. Dong, B. Xie, F. Huang, Stretchable transparent electrodes for conformable wearable organic photovoltaic devices. *npj Flex. Electron.* **5**, 31 (2021)

10. Y. Khan, D. Han, A. Pierre, J. Ting, X. Wang, C. M. Lochner, G. Bovo, N. Yaacobi-Gross, C. Newsome, R. Wilson, A. C. Arias, A flexible organic reflectance oximeter array. *Proc. Natl. Acad. Sci. U.S.A.* **115**, E11015–E11024 (2018).

11. C. M. Lochner, Y. Khan, A. Pierre, A. C. Arias, All-organic optoelectronic sensor for pulse oximetry. *Nat. Commun.* **5**, 5745 (2014).

12. C. Fuentes-Hernandez, W. F. Chou, T. M. Khan, L. Diniz, J. Lukens, F. A. Larrain, V. A. Rodriguez-Toro, B. Kippelen, Large-area low-noise flexible organic photodiodes for detecting faint visible light. *Science* **370**, 698–701 (2020).

13. G. Simone, D. Tordera, E. Delvitto, B. Peeters, A. J. J. M. van Breemen, S. C. J. Meskers, R. A. J. Janssen, G. H. Gelinck, High-accuracy photoplethysmography array using near-infrared organic photodiodes with ultralow dark current. *Adv. Opt. Mater.* **8**, 1901989 (2020).

14. Z. Wu, W. Yao, A. E. London, J. D. Azoulay, T. N. Ng, Temperature-dependent detectivity of near-infrared organic bulk heterojunction photodiodes. *ACS Appl. Mater. Interfaces* **9**, 1654–1660 (2017).

15. J. Simões, T. Dong, Z. Yang, Non-fullerene acceptor organic photodetector for skin-conformable photoplethysmography applications. *Adv. Mater. Interfaces* **9** 2101897 (2022).

16. B. R. Sutherland, A. K. Johnston, A. H. Ip, J. Xu, V. Adinolfi, P. Kanjanaboos, E. H. Sargent, Sensitive, fast, and stable perovskite photodetectors exploiting interface engineering. *ACS Photonics* **2**, 1117–1123 (2015).

17. B. Yang, F. Zhang, J. Chen, S. Yang, X. Xia, T. Pullerits, W. Deng, K. Han, Ultrasensitive and fast all-inorganic perovskite-based photodetector via fast carrier diffusion. *Adv. Mater.* **29**, 1703758 (2017).

18. V. Adinolfi, O. Ouellette, M. I. Saidaminov, G. Walters, A. L. Abdelhady, O. M. Bakr, E. H. Sargent, Fast and sensitive solution-processed visible-blind perovskite UV photodetectors. *Adv. Mater.* **28**, 7264–7268 (2016).

19. F. Guo, Z. Xiao, J. Huang, Fullerene photodetectors with a linear dynamic range of 90 dB enabled by a cross-linkable buffer layer. *Adv. Opt. Mater.* **1**, 289–294 (2013).

20. J. Liu, J. Jiang, S. Wang, T. Li, X. Jing, Y. Liu, Y. Wang, H. Wen, M. Yao, X. Zhan, L. Shen, Fast response organic tandem photodetector for visible and near-infrared digital optical communications. *Small* **17**, 2101316 (2021).

21. J. Lee, S. J. Ko, H. Lee, J. Huang, Z. Zhu, M. Seifrid, J. Vollbrecht, V. V. Brus, A. Karki, H. Wang, K. Cho, T. Q. Nguyen, G. C. Bazan, Side-chain engineering of nonfullerene acceptors for near-infrared organic photodetectors and photovoltaics. *ACS Energy Lett.* **4**, 1401–1409 (2019).

22. C. Xu, P. Liu, C. Feng, Z. He, Y. Cao, Organic photodetectors with high detectivity for broadband detection covering UV-vis-NIR. *J. Mater. Chem. C.* **10**, 5787–5796 (2022).

23. W. Yang, W. Qiu, E. Georgitzikis, E. Simoen, J. Serron, J. Lee, I. Lieberman, D. Cheyns, P. Malinowski, J. Genoe, H. Chen, P. Heremans, Mitigating dark current for high-performance near-infrared organic photodiodes via charge blocking and defect passivation. *ACS Appl. Mater. Interfaces* **13**, 16766–16774 (2021).

24. R. Ollearo, J. Wang, M. J. Dyson, C. H. L. Weijtens, M. Fattori, B. T. Van Gorkom, A. J. J. M. Van Breemen, S. C. J. Meskers, R. A. J. Janssen, G. H. Gelinck, Ultralow dark current in near-infrared perovskite photodiodes by reducing charge injection and interfacial charge generation. *Nat. Commun.* **12**, 7277 (2021).

25. A. Moeini, L. Martínez-Sarti, K. P. S. Zanoni, M. Sessolo, D. Tordera, H. J. Bolink, Semitransparent near-infrared Sn–Pb hybrid perovskite photodetectors. *J. Mater. Chem. C.* **10**, 13878–13885 (2022).

26. X. Xu, C. C. Chueh, P. Jing, Z. Yang, X. Shi, T. Zhao, L. Y. Lin, A. K. Y. Jen, High-performance near-IR photodetector using low-bandgap $MA_{0.5}FA_{0.5}Pb_{0.5}Sn_{0.5}I_3$ perovskite. *Adv. Funct. Mater.* **27**, 1701053 (2017).

27. W. Wang, D. Zhao, F. Zhang, L. Li, M. Du, C. Wang, Y. Yu, Q. Huang, M. Zhang, L. Li, J. Miao, Z. Lou, G. Shen, Y. Fang, Y. Yan, Highly sensitive low-bandgap perovskite photodetectors with response from ultraviolet to the near-infrared region. *Adv. Funct. Mater.* **27**, 1703953 (2017).

28. S. J. Lim, D. S. Leem, K. B. Park, K. S. Kim, S. Sul, K. Na, G. H. Lee, C. J. Heo, K. H. Lee, X. Bulliard, R. I. Satoh, T. Yagi, T. Ro, D. Im, J. Jung, M. Lee, T. Y. Lee, M. G. Han, Y. W. Jin, S. Lee,

Organic-on-silicon complementary metal-oxide-semiconductor colour image sensors. *Sci. Rep.* **5**, 7708 (2015).

29. K. H. Lee, G. H. Lee, D. S. Leem, J. Lee, J. W. Chung, X. Bulliard, H. Choi, K. B. Park, K. S. Kim, Y. W. Jin, S. Lee, S. Y. Park, Dynamic characterization of green-sensitive organic photodetectors using nonfullerene small molecules: Frequency response based on the molecular structure. *J. Phys. Chem. C.* **118**, 13424–13431 (2014).

30. D. M. Lyons, A. Armin, M. Stolterfoht, R. C. R. Nagiri, R. D. Jansen-Van Vuuren, B. N. Pal, P. L. Burn, S. C. Lo, P. Meredith, Narrow band green organic photodiodes for imaging. *Org. Electron.* **15**, 2903–2911 (2014).

31. L. Gao, C. Ge, W. Li, C. Jia, K. Zeng, W. Pan, H. Wu, Y. Zhao, Y. He, J. He, Z. Zhao, G. Niu, X. Guo, F. P. G. de Arquer, E. H. Sargent, J. Tang, Flexible filter-free narrowband photodetector with high gain and customized responsive spectrum. *Adv. Funct. Mater.* **27**, 1702360 (2017).

32. J. Li, J. Wang, J. Ma, H. Shen, L. Li, X. Duan, D. Li, Self-trapped state enabled filterless narrowband photodetections in 2D layered perovskite single crystals. *Nat. Commun.* **10** 806 (2019).

33. A. Armin, R. D. Jansen-Van Vuuren, N. Kopidakis, P. L. Burn, P. Meredith, Narrowband light detection via internal quantum efficiency manipulation of organic photodiodes. *Nat. Commun.* **6** 6343 (2015).

34. Q. Lin, A. Armin, P. L. Burn, P. Meredith, Filterless narrowband visible photodetectors. *Nat. Photonics.* **9**, 687–694 (2015).

35. J. Wang, S. Xiao, W. Qian, K. Zhang, J. Yu, X. Xu, G. Wang, S. Zheng, S. Yang, Self-driven perovskite narrowband photodetectors with tunable spectral responses. *Adv. Mater.* **33**, 200555 (2021).

36. T. J. K. Brenner, Y. Vaynzof, Z. Li, D. Kabra, R. H. Friend, C. R. McNeill, White-light bias external quantum efficiency measurements of standard and inverted P3HT:PCBM photovoltaic cells. *J. Phys. D Appl. Phys.* **45**, 415101 (2012).

37. J. Liu, Y. Zou, B. Huang, Y. Gu, Y. Yang, Z. Han, Y. Zhang, X. Xu, H. Zeng, Sensitively switchable visible/infrared multispectral detection and imaging based on a tandem perovskite device. *Nanoscale* **12**, 20386–20395 (2020).

38. L. Li, Y. Deng, C. Bao, Y. Fang, H. Wei, S. Tang, F. Zhang, J. Huang, Self-filtered narrowband perovskite photodetectors with ultrafast and tuned spectral response. *Adv. Opt. Mater.* **5**, 1700672 (2017).

39. Y. Zhang, Z. Qin, X. Huo, D. Song, B. Qiao, S. Zhao, High-performance near-infrared photodetectors based on the synergy effect of short wavelength light filter and long wavelength response of a perovskite/polymer hybrid structure. *ACS Appl. Mater. Interfaces* **13**, 61818–61826 (2021).

40. B. Xie, R. Xie, K. Zhang, Q. Yin, Z. Hu, G. Yu, F. Huang, Y. Cao, Self-filtering narrowband high performance organic photodetectors enabled by manipulating localized Frenkel exciton dissociation. *Nat. Commun.* **11**, 2871 (2020).

41. B. Siegmund, A. Mischok, J. Benduhn, O. Zeika, S. Ullbrich, F. Nehm, M. Böhm, D. Spoltore, H. Fröb, C. Körner, K. Leo, K. Vandewal, Organic narrowband near-infrared photodetectors based on intermolecular charge-transfer absorption. *Nat. Commun.* **8**, 15421 (2017).

42. Z. Lan, L. Cai, D. Luo, F. Zhu, Narrowband near-infrared perovskite/polymer hybrid photodetectors. *ACS Appl. Mater. Interfaces* **13**, 981–988 (2021).

43. J. Zhao, X. Wang, Y. Xu, Y. Pan, Y. Li, J. Chen, Q. Li, X. Zhang, Z. Zhu, Z. Zhao, E. E. Elemike, D. C. Onwudiwe, B. S. Bae, S. Bin Shafie, W. Lei, Electrically modulated near-infrared/visible light dual-mode perovskite photodetectors. *ACS Appl. Mater. Interfaces* **13**, 25824–25833 (2022).

44. S. Qiao, Y. Liu, J. Liu, G. Fu, S. Wang, High-responsivity, fast, and self-powered narrowband perovskite heterojunction photodetectors with a tunable response range in the visible and near-infrared region. *ACS Appl. Mater. Interfaces* **13**, 34625–34636 (2021).

45. Y. S. Shin, Y. J. Yoon, J. Heo, S. Song, J. W. Kim, S. Y. Park, H. W. Cho, G. H. Kim, J. Y. Kim, Functionalized PFN-X (X = Cl, Br, or I) for balanced charge carriers of highly efficient blue light-emitting diodes. *ACS Appl. Mater. Interfaces* **12**, 35740–35747 (2020).

46. Q. Lin, A. Armin, D. M. Lyons, P. L. Burn, P. Meredith, Low noise, IR-blind organohalide perovskite photodiodes for visible light detection and imaging. *Adv. Mater.* **27**, 2060–2064 (2015).

47. Y. Wang, J. Kublitski, S. Xing, F. Dollinger, D. Spoltore, J. Benduhn, K. Leo, Narrowband organic photodetectors - towards miniaturized, spectroscopic sensing. *Mater. Horiz.* **9**, 220–251 (2022).

48. W. Zhang, C. Sun, I. Angunawela, L. Meng, S. Qin, L. Zhou, S. Li, H. Zhuo, G. Yang, Z. G. Zhang, H. Ade, Y. Li, 16.52% efficiency all-polymer solar cells with high tolerance of the photoactive layer thickness. *Adv. Mater.* **34**, 2108749 (2022).

49. B. H. Jiang, Y. P. Wang, C. Y. Liao, Y. M. Chang, Y. W. Su, R. J. Jeng, C. P. Chen, Improved blend film morphology and free carrier generation provide a high-performance ternary polymer solar cell. *ACS Appl. Mater. Interfaces* **13**, 1076–1085 (2021).

50. J. Yuan, Y. Zhang, L. Zhou, G. Zhang, H. L. Yip, T. K. Lau, X. Lu, C. Zhu, H. Peng, P. A. Johnson, M. Leclerc, Y. Cao, J. Ulanski, Y. Li, Y. Zou, Single-junction organic solar cell with over 15% efficiency using fused-ring acceptor with electron-deficient core. *Joule* **3**, 1140–1151 (2019).

51. N. F. Watson, M. S. Badr, G. Belenky, D. L. Bliwise, O. M. Buxton, D. Buysse, D. F. Dinges, J. Gangwisch, M. A. Grandner, C. Kushida, R. K. Malhotra, J. L. Martin, S. R. Patel, S. F. Quan, E. Tasali, M. Twery, J. B. Croft, E. Maher, J. A. Barrett, S. M. Thomas, J. L. Heald, Joint consensus statement of the American Academy of Sleep Medicine and Sleep Research Society on the recommended amount of sleep for a healthy adult: Methodology and discussion. *J. Clin. Sleep Med.* **11**, 931–952 (2015).

52. S. Xing, J. Kublitski, C. Hänisch, L. C. Winkler, T.-y. Li, H. Kleemann, J. Benduhn, K. Leo, Photomultiplication-type organic photodetectors for near-infrared sensing with high and bias-independent specific detectivity. *Adv. Sci.* **9**, 2105113 (2022).

53. Y. Wang, Y. Liu, S. Cao, J. Wang, A review on solution-processed perovskite/organic hybrid photodetectors. *J. Mater. Chem. C.* **9**, 5302–5322 (2021).

54. Z. Lan, M.-H. Lee, F. Zhu, Recent advances in solution-processable organic photodetectors and applications in flexible electronics. *Adv. Intell. Syst.* **4**, 2100167 (2022).

55. E. Allado, M. Poussel, J. Renno, A. Moussu, O. Hily, M. Temperelli, E. Albuisson, B. Chenuel, Remote photoplethysmography is an accurate method to remotely measure respiratory rate: A hospital-based trial. *J. Clin. Med.* **11**, 3647 (2022).

56. J. Wang, K. Datta, J. Li, M. A. Verheijen, D. Zhang, M. M. Wienk, R. A. J. Janssen, Understanding the film formation kinetics of sequential deposited narrow-bandgap Pb–Sn hybrid perovskite films. *Adv. Energy Mater.* **10**, 2000566 (2020).

57. M. Mulato, C. M. Hong, S. Wagner, Size and etching effects on the reverse current of a-Si:H p-i-n diodes. *J. Electrochem. Soc.* **150**, G735 (2003).

58. A. J. J. M. van Breemen, R. Ollearo, S. Shanmugam, B. Peeters, L. C. J. M. Peters, R. L. van de Ketterij, I. Katsouras, H. B. Akkerman, C. H. Frijters, F. Di Giacomo, S. Veenstra, R. Andriessen, R. A. J. Janssen, E. A. Meulenkamp, G. H. Gelinck, A thin and flexible scanner for fingerprints and documents based on metal halide perovskites. *Nat. Electron* **4**, 818–826 (2021).

59. Q. Liu, S. Zeiske, X. Jiang, D. Desta, S. Mertens, S. Gielen, R. Shanivarasanthe, H. G. Boyen, A. Armin, K. Vandewal, Electron-donating amine-interlayer induced n-type doping of polymer: Nonfullerene blends for efficient narrowband near-infrared photo-detection. *Nat. Commun.* **13**, 5194 (2022).

60. C. Kaiser, K. S. Schellhammer, J. Benduhn, B. Siegmund, M. Tropiano, J. Kublitski, D. Spoltore, M. Panhans, O. Zeika, F. Ortmann, P. Meredith, A. Armin, K. Vandewal, Manipulating the charge transfer absorption for narrowband light detection in the near-infrared. *Chem. Mater.* **31**, 9325–9330 (2019).

61. L. Shen, Y. Zhang, Y. Bai, X. Zheng, Q. Wang, J. Huang, A filterless, visible-blind, narrow-band, and near-infrared photodetector with a gain. *Nanoscale* **8**, 12990–12997 (2016).

62. M. Liu, J. Wang, Z. Zhao, K. Yang, P. Durand, F. Ceugniet, G. Ulrich, L. Niu, Y. Ma, N. Leclerc, X. Ma, L. Shen, F. Zhang, Ultra-narrow-band NIR photomultiplication organic photodetectors based on charge injection narrowing. *J. Phys. Chem. Lett.* **12**, 2937–2943 (2021).

63. W. Wang, M. Du, M. Zhang, J. Miao, Y. Fang, F. Zhang, Organic photodetectors with gain and broadband/narrowband response under top/bottom illumination conditions. *Adv. Opt. Mater.* **6**, 1800249 (2018).

64. W. Cheng, W. Tian, F. Cao, L. Li, Self-powered bifunctional perovskite photodetectors with both broadband and narrowband photoresponse. *InfoMat* **4**, e12348 (2022).

65. J. Miao, F. Zhang, M. Du, W. Wang, Y. Fang, Photomultiplication type organic photodetectors with broadband and narrowband response ability. *Adv. Opt. Mater.* **6**, 1800001 (2018).
